# Two-dimensional fluoroscopic instrument tracking-assisted prone lateral lumbar interbody fusion

**DOI:** 10.3171/2026.4.FOCVID2643

**Published:** 2026-07-01

**Authors:** Favour C. Ononogbu-Uche, Isabella Decker, Jordan Hatfield, Muhammad M. Abd-El-Barr

**Affiliations:** Department of Neurosurgery, Duke University, Durham, North Carolina

**Keywords:** fluoroscopy, instrument tracking, interbody fusion, lumbar spine, prone lateral, two-dimensional

## Abstract

A 55-year-old male with prior L5–S1 posterior lumbar interbody fusion developed adjacent segment degeneration with severe stenosis at L3–4 and L4–5 and underwent L3–5 prone lateral lumbar interbody fusion with extension of fusion using 2D fluoroscopic instrument tracking (2D-FIT). The TrackX system used standard anteroposterior and lateral fluoroscopic images to assist with trajectory planning, docking, retractor placement, disc preparation, trialing, and cage delivery. Simultaneous percutaneous pedicle screw fixation was performed by a co-surgeon to improve single-position efficiency. There were no intraoperative complications, neurological examinations remained stable, and hardware alignment was satisfactory. The patient was discharged home on postoperative day 3. Two-dimensional FIT helps improve efficiency without compromising safety.

The video can be found here: https://stream.cadmore.media/r10.3171/2026.4.FOCVID2643

## Transcript

### 0:27 Prone Lateral Lumbar Interbody Fusion.

The prone lateral lumbar interbody fusion (PL-LIF) has been well established as a superior alternative to its regular lateral counterpart due to its single-position nature offering an efficient transition from lateral to posterior work.^1^ However, some persistent challenges include intraoperative localization, trajectory control, and the use of repeated confirmatory x-ray shots.^1^ Two-dimensional fluoroscopic instrument tracking (2D-FIT) addresses these limitations by allowing surgeons to plan trajectory and decrease uncertainty during docking, retraction, disc prep, and implant delivery.^2^ Importantly, tracking is not limited by single instruments and allows for simultaneous screw placement with a co-surgeon.^2^

### 1:10 Objectives.

Therefore, the objectives of this video are to demonstrate the setup and intraoperative use of 2D fluoroscopic instrument tracking during prone lateral lumbar interbody fusion, show how tracking enhances safe instrument localization and workflow consistency using standard anteroposterior (AP) and lateral fluoroscopy, and highlight how the technique facilitates lateral access while allowing simultaneous posterior pedicle screw fixation.

### 1:36 Two-Dimensional Fluoroscopic Instrument Tracking.

The 2D-FIT technology used in this surgery was TrackX. It requires the use of frame attachments for the C-arm containing navigational spheres, and similar navigational clips on required surgical instruments. Tracking clips (green, yellow, and purple) are attached directly to the instrument being used, and the color of the clip corresponds to the color on the display monitor. The reference frame (blue) is attached directly onto the source and detector poles of the C-arm. After acquisition of AP and lateral shots, the system automatically registers those images and then continuously overlays tracked instrument motion onto the saved views in real time. These attachments allow for real-time tracking of instrument overlay on saved AP and lateral fluoroscopic images that are taken intraoperatively as the surgeon operates or suspects anatomical mismatch. By displaying both AP and lateral dimensions simultaneously, 2D-FIT allows the surgeon to know where instruments are always during the procedure. After a new image is taken, any movement in the instrumentation will be continuously updated and displayed in real-time.^2^ Due to the system’s fluoroscopy centricity, it does not require a preoperative CT scan and can be used to track multiple instruments throughout the operation. For proper use, the system requires input from two orthogonal fluoroscopic images, registration, and instruments to be tracked. It also requires high-quality images, stable patient position, and planned reregistration points during the surgery.

### 3:07 Clinical Presentation and Surgical Plan.

A 55-year-old man with a history of L5–S1 posterior lumbar interbody fusion performed more than 15 years earlier presented with chronic low back and bilateral leg pain lasting more than 1 year, which had progressed to constant severe pain requiring a mobility aid. He had exhausted medical management, and epidural injections were no longer providing relief. He reported low back pain with radiation down the posterior aspect of both legs. Preoperative lumbar spine MRI demonstrated adjacent segment degeneration above the prior L5–S1 fusion, with severe stenosis at L3–4 and L4–5 due to diffuse disc bulging and ligamentum flavum hypertrophy. After discussion of treatment options, the patient agreed to undergo L3–5 PL-LIF with extension of fusion.

### 4:08 Surgical Procedure.

After initial steps, such as general anesthesia induction, administration of perioperative antibiotics, and endotracheal intubation, the patient was placed in the prone position. Two-dimensional FIT is utilized to plan the lateral trajectory to the L3–4 and L4–5 disc space with a single incision. Incisions are planned posteriorly to expose the prior instrumentation and prepare for extension of fusion. These photographs show the operating room (OR) setup with 2D-FIT. The patient is prone with the lateral corridor exposed, instruments ready with colored navigational clips attached (green and blue), and a draped C-arm positioned for AP and lateral imaging with blue navigational frame, while the monitor displays real-time tracking overlay for localization and trajectory guidance. This set of videos depicts the visualization of the surgical instrument on the AP and lateral images over the disc space, prior to Jamshidi placement into the L4 pedicle. Next, the Jamshidi is inserted into the left L4 pedicle to serve as an internal reference to compare future images to. Further trajectory planning and navigation is performed to allow for confirmation that surgeons are in the appropriate position in the disc space prior to docking. Afterward, the retractor is positioned over the disc space, and a K-wire is introduced into the disc space to serve as an anchor. The dilator is opened to reveal the disc space. Shown here is the interbody work phase before disc preparation. The patient is prone with the lateral retractor docked and mounted, and the draped C-arm is positioned for standard AP and lateral views as the monitor continues to display the real-time tracking overlay on the stored orthogonal images. Here we can once again see colored navigational clips (green on left and orange on right) on the navigated tools, and the blue navigational frame on the C-arm. Next, various instruments for disc space preparation are utilized including a curette, a shaver, a box cutter, and trialing. These can be navigated as well as the entire disc preparation can be performed with navigated instruments. After the interbody cage is placed at a particular level, the surgeon can then reposition for other levels and repeat steps as needed. Here, the primary surgeon places interbodies before pedicle screws to optimize tracking visualization and simplify lateral trajectory planning by minimizing additional posterior hardware in the fluoroscopic field. After interbody placement, we obtain repeat AP and lateral images, reregister as needed to confirm persistent accuracy, and then proceed with the next interbody or posterior pedicle screw fixation.

### 6:55 Simultaneous Percutaneous Pedicle Screw Placement.

The key step to highlight is the placement of simultaneous percutaneous pedicle screws. This maximizes the efficiency of single position to allow immediate posterior instrumentation. The two-surgeon parallelization allows for reduced idle time and total operating room time. It also allows for specific side or level assignment, shared fluoroscopic cadence, and standardized callouts.^2,3^

### 7:23 Workflow Overview.

The surgical workflow while using 2D-FIT includes a stepwise loop of obtaining an image, registering the image and an instrument, tracking during a task, confirming with a new image, and reregistering when necessary.^2^ Triggers for reregistration include repositioning of the retractor, anatomy shifts, a new view, or any mismatch with the displayed overlay. This allows for a predictable cadence of imaging checks with less images required to localized anatomy and instrumentation, as opposed to unnecessary repeated shots.^2,4^

### 7:51 Postoperative Course.

There were no intraoperative complications. The case lasted 4 hours, and there were 200 mL of blood loss. Postoperatively, the patient remained stable in the postanesthesia care unit and was then transferred to the step-down unit, where serial neurological examinations were performed. His diet was advanced, and pain control was transitioned to oral medications. The urinary catheter and surgical drain were removed without issue. Postoperative radiographs demonstrated good hardware alignment. After evaluation by physical therapy and occupational therapy, he was discharged home on postoperative day (POD) 3 with home physical therapy and occupational therapy.

### 8:26 Safety Checks, Limitations, Comparison With 3D Navigation, and Bottom Line.

Tracking complements fluoroscopy, anatomical landmarks, tactile feedback, and neuromonitoring when used, and reimaging should be performed after retractor repositioning, anatomical shift, acquisition of a new view, or any overlay mismatch.^2,4^ Limitations include registration drift, dependence on image quality, a learning curve, and the continued need for confirmation shots, although often less frequent than with standard fluoroscopic guidance.^4,5^

Two-dimensional FIT integrates directly with standard AP and lateral C-arm imaging to provide real-time instrument overlay, which fits naturally into an efficient single-position prone lateral workflow without requiring a 3D intraoperative spin or a preoperative CT, and it supports tracking of multiple instruments.^1,2^ By contrast, 3D navigation platforms such as O-arm with Stealth provide a true 3D view and have reported high pedicle screw placement accuracy. However, 3D navigation typically adds bulky equipment and acquisition steps that can increase workflow overhead and interruptions compared with C-arm–based approaches.^2,6^ Overall, 2D-FIT provides the greatest value during docking, retractor placement, trialing, and cage delivery, and it pairs well with single-position efficiency by enabling parallel pedicle screw placement, helping improve efficiency without compromising safety.^2,3^

## Disclosures

M. Abd-El-Barr reported consulting for TrackX, Brainlab, and Arthrex outside the submitted work.

## Author Contributions

Primary surgeon: Abd-El-Barr. Assistant surgeon: Hatfield. Editing and drafting the video and abstract: all authors. Critically revising the work: Abd-El-Barr, Ononogbu-Uche, Hatfield. Reviewed submitted version of the work: Abd-El-Barr, Decker. Approved the final version of the work on behalf of all authors: Abd-El-Barr.

## Supplemental Information

### Patient Informed Consent

The necessary patient informed consent was obtained in this study.

## References

[1] Pimenta L Amaral R Taylor W The prone transpsoas technique: preliminary radiographic results of a multicenter experience Eur Spine J 2021 30 1 108 113 10.1007/s00586-020-06471-y 32472346

[2] Srinivasan ES Hamouda F Gnaedinger AG Instrument tracking for prone lateral surgery World Neurosurg 2023 169 42 10.1016/j.wneu.2022.10.119 36336269

[3] Wang TY Hamouda F Mehta VA Effect of instrument navigation on C-arm radiation and time during spinal procedures: a clinical evaluation Int J Spine Surg 2020 14 3 375 381 10.14444/7049 32699760 PMC7343269

[4] Rawicki N Dowdell JE Sandhu HS Current state of navigation in spine surgery Ann Transl Med 2021 9 1 85 10.21037/atm-20-1335 33553378 PMC7859779

[5] Wang TY Mehta VA Sankey EW Lavoie S Abd-El-Barr MM Yarbrough CK Operative time and learning curve between fluoroscopy-based instrument tracking and robot-assisted instrumentation for patients undergoing minimally invasive transforaminal lumbar interbody fusion (MIS-TLIF) Clin Neurol Neurosurg 2021 206 106698 10.1016/j.clineuro.2021.106698 34030076

[6] Patil S Lindley EM Burger EL Yoshihara H Patel VV Pedicle screw placement with O-arm and stealth navigation Orthopedics 2012 35 1 e61 e65 10.3928/01477447-20111122-15 22229616

